# Prevalence and characterization of ciprofloxacin-resistant *Salmonella enterica* spp. isolated from food animals during 2010–2023 in South Korea

**DOI:** 10.1080/01652176.2025.2473733

**Published:** 2025-03-17

**Authors:** Md. Sekendar Ali, Hee-Seung Kang, Bo-Youn Moon, Ye-Eun Heo, Min Young Kim, Ji-Hyun Choi, Yu-Jeong Hwang, Ji-In Kim, Yeon-Hee Lee, Jae-Myung Kim, Suk-Kyung Lim

**Affiliations:** Department of Animal and Plant Quarantine Agency, Bacterial Disease Division, Animal and Plant Quarantine Agency, Gimcheon-si, Republic of Korea

**Keywords:** *Salmonella*, food animals, ciprofloxacin resistance, mutation, STs, pulsotypes

## Abstract

We isolated 6,561 *Salmonella* strains from food animals, cattle (*n* = 217), pigs (*n *= 1526), chickens (*n* = 3942), and ducks (*n* = 876). Isolates were evaluated for antimicrobial sensitivity, mutations in quinolone resistance determination regions (QRDRs), and plasmid-mediated quinolone resistance (PMQR) genes. Clonal relationship and genetic diversity were assessed by multi-locus sequence typing (MLST) and pulsed-field gel electrophoresis (PFGE). Overall, 3.1% of isolates exhibited resistance to ciprofloxacin. Commonly identified mutations in QRDRs were S83F, D87N, and D87G in *gyrA*; T57S and S80I in *parC*; and L416F in *parE*. Furthermore, mutations differed by serotypes. In *S.* Albany, S83F mutation in *gyrA* and T57S in *parC* were prevalent, while in *S.* Kentucky, S83F and D87N in *gyrA*, T57S and S80I in *parC*; and in *S.* Indiana, S83F and D87G in *gyrA*, T57S and S80R in *parC*, and L416F in *parE* were common. Amongst PMQRs, *qnrS* was mainly observed in *S.* Albany, *aac(6′)-Ib-cr* in *S.* Indiana, and *qnrB1* in *S.* Albany. Among STs, ST198 *S.* Kentucky was predominant, followed by ST292 *S.* Albany and ST17 *S.* Indiana. Of 26 pulsotypes, KX1KA1 was mainly identified in *S.* Kentucky, AX1AA1 in *S.* Albany, and IX1IA1 in *S.* Indiana. Taken together, ciprofloxacin-resistant *Salmonella* can pose health hazards to humans and other animals.

## 1. Introduction

*Salmonella* is one of the most common pathogens causing foodborne illness in humans globally. According to the global burden of disease study, approximately 535,000 cases of non-typhoidal human salmonellosis were detected in 2017 worldwide (Stanaway et al. 2019). In Korea, there were an average of 857 reported cases per year from 2009 to 2020 of individuals infected with *Salmonella* (Koh et al. 2022). Although non-typhoidal salmonellosis usually recovers spontaneously, suitable antimicrobial therapy is essential for severe cases, particularly those with compromised immune systems, pediatrics, and geriatrics (Sánchez-Vargas et al. 2011). Nevertheless, numerous countries, including developing and developed ones, have reported antimicrobial-resistant *Salmonella* infections, resulting in mortality and morbidity (Majowicz et al. 2010; Kariuki et al. 2015; Parisi et al. 2018). The use of antimicrobials in both humans and food animals leads to the spread of antimicrobial resistance; consequently, the effectiveness of the most commonly used antimicrobials, such as penicillin, tetracycline, and chloramphenicol, has become limited (Kuang et al. 2018). As a result, fluoroquinolones, including ciprofloxacin, have become a preferred treatment for invasive salmonellosis due to their effectiveness in treating a wide range of infections (Cuypers et al. 2018). However, the widespread use of quinolone and fluoroquinolone inevitably triggers the development of antimicrobial resistance with novel resistance mechanisms (Li et al. 2018).

The prevalence of ciprofloxacin-resistant *Salmonella* isolated from food animals has been described worldwide (Hur et al. 2012). Moreover, numerous studies on the occurrence of ciprofloxacin resistance in *Salmonella* isolates from humans have been reported in China (Kuang et al. 2018), Poland (Wołkowicz et al. 2021), and Korea (Lee et al. 2021). In addition, it was shown that ciprofloxacin resistance can be transmitted to humans through direct contact or consumption of *Salmonella*-contaminated meat of food-producing animals (Bulut 2014).

Recently, the prevalence of fluoroquinolone-resistant *Salmonella* has been augmented in food animals in Korea (Kim et al. 2019; Moon et al. 2021; Zhang et al. 2021). Understanding the phenotypic and genotypic characteristics of *Salmonella* in food animals is essential for ascertaining the potential risk to humans. Therefore, the purpose of this study was to determine the prevalence at the national level and molecular characteristics of ciprofloxacin-resistant *Salmonella* spp. isolated from food animals during 2010–2023 in South Korea.

## 2. Materials and methods

### 2.1. Bacterial isolation and identification

*Salmonella* isolation, identification, and serotyping were carried out according to the previously delineated method (Ali et al. 2023). The *Salmonella* strains were obtained from feces and carcasses of food-producing animals (cattle, pigs, chickens, and ducks) from 16 laboratories/centers participating in the Korean Veterinary Antimicrobial Resistance Monitoring System (KVARS) between 2010 and 2023. The isolation process involves the sample pre-enrichment in buffered peptone water followed by incubation in a modified semisolid Rappaport Vassiliadis medium (MSRV: Becton Dickinson, CA, USA). The diffused regions at MSRV were cultured using CHROMagar (Merck, Darmstadt, Germany), and the colonies were identified by matrix-assisted laser desorption ionization-time-of-flight mass spectrometry (Biomerieux, Marcy L’Etoile, France). *Salmonella* serotype was determined using polymerase chain reaction (PCR) (Ranieri et al. 2013; Mechesso et al. 2022) and finally confirmed by the classic White-Kauffman-Le Minor scheme (Grimont and Weill 2007). One isolate from each sample was used for subsequent analysis. However, we do not have information on the history of antimicrobial usage in food-producing animals considered for this investigation.

### 2.2. Antimicrobial susceptibility testing

The antimicrobial susceptibility of the isolates was assessed by the broth microdilution method (Ali et al. 2024) using the Sensititre^TM^ panel KRNV6F (Thermo Fischer Scientific, Waltham, MA, USA) based on the manufacturer’s protocol. The obtained minimum inhibitory concentrations (MICs) were interpreted according to the guidelines provided by the Clinical and Laboratory Standards Institute (CLSI) (2023). The MIC ≥4 µg/mL of ciprofloxacin was set as a threshold value to indicate a high level of ciprofloxacin resistance (Raveendran et al. 2008). The high-level ciprofloxacin-resistant isolates were further characterized using molecular techniques.

### 2.3. Mechanisms of ciprofloxacin resistance

Genes encoding for DNA gyrase (*gyrA* and *gyrB*) and topoisomerase IV (*parC* and *parE*) in quinolone resistance determining regions (QRDRs) were performed using multiplex PCR. The PCR products were subjected to sequencing using an automated ABI Prism 3700 analyzer (Applied Biosystems, Foster, CA, USA). The primers and PCR conditions are summarized in the Supplementary Table S1. We used the Basic Local Alignment Search Tool (BLAST) to identify gene mutations in QRDRs. We compared the sequences with those available in the GenBank nucleotide database at the National Center for Biotechnology Information (http://www.ncbi.nlm.nih.gov/BLAST). The detection of plasmid-mediated quinolone resistance (PMQR) genes (*qnrB1, qnrS,* and *aac(6′)-Ib-cr*) was performed by PCR using the previously described conditions and primers (Table S1).

### 2.4. Mechanism of third-generation cephalosporin resistance

The double-disc synergy was performed to detect the presence of extended-spectrum β-lactamase (ESBL) genes among the ciprofloxacin-resistant *Salmonella* using cefotaxime-cefotaxime/clavulanic acid discs, following the guidelines provided by the Clinical and Laboratory Standards Institute (CLSI) (2023). The PCR was conducted using previously described primers to identify the presence of ESBL (*bla*_CTX-M_) genes in cefotaxime-resistant *Salmonella* isolates (Na et al. 2020). First, we detected the CTX-M gene using a universal primer, followed by the detection of the CTX-M-9 group gene using *bla*_CTX-M_ group-specific primers. Then, we performed the PCR and sequencing using primers covering the whole CTX-M-9 group gene. The sequencing of PCR products was performed using an ABI3730XL DNA sequencing analyzer (Solgnet Daejeon, South Korea) to identify the *bla*_CTX-M_ by searching the homologous sequence in the GenBank database utilizing the Basic Local Alignment Search Tool (BLAST) on the National Center for Biotechnology Information website (http://www.ncbi.nlm.gov/BLAST). The primers and PCR conditions are detailed in Table S1.

### 2.5. Multi-locus sequence typing (MLST) and pulsed-field gel electrophoresis (PFGE)

The clonal relationship of *Salmonella* isolates was determined using MLST, following the previous method (Kidgell et al. 2002). A total of seven housekeeping genes (*aroC*, *dnaN*, *hemD*, *hisD*, *purE*, *sucA*, and* thrA*) were amplified and sequenced in this classical approach. The allelic profiles and sequence types (STs) for *Salmonella* spp. were identified using the online MLST database (http://pubmlst.org/organisms/Salmonella-spp). The genetic diversity of *Salmonella* isolates was evaluated using PFGE of genomic DNA digested with the enzymes *Xba*I and *Avr*II (TaKaRa Bio, Inc., Shiga, Japan) (Ali et al. 2023). The PFGE band profiles were assessed by Bionumerics software (version 5.1), and the level of similarity was evaluated using the unweighted pair-group technique with an algorithm-based arithmetic average and Dice similarity index.

## 3. Results

### 3.1. Prevalence of Salmonella

A total of 6561 *Salmonella* strains have been isolated from feces and carcasses of food-producing animals (Table 1). Overall, ciprofloxacin resistance was found in 3.1% of the isolates. The resistance rate was varied by different year groups. The level of resistance was high during 2010–2012 but was lower in the period of 2016–2018. However, the proportion of isolates that showed MIC ≤0.12 µg/mL decreased from 64.4% to 37% in 2010–2012 and 2022–2023. Furthermore, isolates that showed high levels of MIC were increased.

**Table 1.** Distribution of ciprofloxacin-resistant *Salmonella* isolates obtained from food animals during 2010–2023 in South Korea.

		Minimum inhibitory concentration (MIC) of ciprofloxacin (µg/mL)									
		≤0.12	0.25	0.5	1	2	4	8	16	>16	≥1
Cattle	2010–2012 (*n* = 75)	80.0 (60)	12.0 (9)	4.0 (3)	4.0 (3)	0 (0)	0 (0)	0 (0)	0 (0)	0 (0)	4.0 (3)
	2013–2015 (*n* = 60)	61.7 (37)	15.0 (9)	16.7 (10)	6.7 (4)	0 (0)	0 (0)	0 (0)	0 (0)	0 (0)	6.7 (4)
	2016–2018 (*n* = 43)	48.8 (21)	9.3 (4)	41.9 (18)	0 (0)	0 (0)	0 (0)	0 (0)	0 (0)	0 (0)	0 (0)
	2019–2021 (*n* = 25)	32.0 (8)	44.0 (11)	24.0 (6)	0 (0)	0 (0)	0 (0)	0 (0)	0 (0)	0 (0)	0 (0)
	2022–2023 (*n* = 14)	71.4 (10)	14.3 (2)	14.3 (2)	0 (0)	0 (0)	0 (0)	0 (0)	0 (0)	0 (0)	0 (0)
	Subtotal (*n* = 217)	62.7 (136)	16.1 (35)	18.0 (39)	3.2 (7)	0 (0)	0 (0)	0 (0)	0 (0)	0 (0)	3.2 (7)
Pigs	2010–2012 (*n* = 305)	73.8 (225)	11.2 (34)	14.1 (43)	1.0 (3)	0 (0)	0 (0)	0 (0)	0 (0)	0 (0)	1.0 (3)
	2013–2015 (*n* = 360)	78.1 (281)	6.9 (25)	14.4 (52)	0.6 (2)	0 (0)	0 (0)	0 (0)	0 (0)	0 (0)	0.6 (2)
	2016–2018 (*n* = 295)	70.5 (208)	11.9 (35)	16.3 (48)	0.7 (2)	0.7 (2)	0 (0)	0 (0)	0 (0)	0 (0)	1.3 (4)
	2019–2021 (*n* = 406)	69.5 (282)	11.8 (48)	14.0 (57)	2.5 (10)	1.7 (7)	0.2 (1)	0.2 (1)	0 (0)	0 (0)	4.6 (19)
	2022–2023 (*n* = 160)	67.5 (108)	12.5 (20)	17.5 (28)	1.9 (3)	0 (0)	0 (0)	0 (0)	0 (0)	0 (0)	1.9 (3)
	Subtotal (*n* = 1526)	72.3 (1104)	10.6 (162)	14.9 (228)	1.3 (20)	0.6 (9)	0.1 (1)	0.1 (1)	0 (0)	0 (0)	2.0 (31)
Chickens	2010–2012 (*n* = 555)	57.1 (317)	22.3 (124)	13.3 (74)	5.6 (31)	1.6 (9)	0 (0)	0 (0)	0 (0)	0 (0)	7.2 (40)
	2013–2015 (*n* = 633)	37.0 (234)	45.2 (286)	13.4 (85)	1.6 (10)	2.2(14)	0.5 (3)	0.2 (1)	0 (0)	0 (0)	4.4 (28)
	2016–2018 (*n* = 692)	36.1 (250)	54.5 (377)	8.2 (57)	0.3 (2)	0.7 (5)	0 (0)	0.1 (1)	0 (0)	0 (0)	1.2 (8)
	2019–2021 (*n* = 1079)	41.2 (445)	33.8 (365)	21.2 (229)	0.6 (6)	0.6 (7)	0.2 (2)	0.8 (9)	0.6 (7)	0.7 (8)	3.6 (39)
	2022–2023 (*n* = 983)	27.6 (271)	56.4 (554)	14.5 (143)	1.0 (10)	0.1 (1)	0 (0)	0.3 (3)	0.1 (1)	0 (0)	1.5 (15)
	Subtotal (*n* = 3942)	38.5 (1517)	43.2 (1704)	14.9 (588)	1.5 (59)	0.9 (36)	0.1 (5)	0.4 (14)	0.2 (8)	0.2 (8)	3.2 (130)
Ducks	2016–2018 (*n* = 94)	78.7 (74)	5.3 (5)	14.9 (14)	0 (0)	0 (0)	0 (0)	0 (0)	0 (0)	1.1 (1)	1.1 (1)
	2019–2021 (*n* = 510)	59.2 (302)	21.0 (107)	15.1 (77)	0.6 (3)	1.6 (8)	1.0 (5)	1.0 (5)	0.2 (1)	0.4 (2)	4.7 (24)
	2022–2023 (*n* = 272)	51.5 (140)	33.5 (91)	9.9 (27)	0.4 (1)	3.0 (8)	0 (0)	0.4 (1)	1.1 (3)	0 (0)	4.8 (13)
	Subtotal (*n* = 876)	58.9 (516)	23.2 (203)	13.5 (118)	0.5 (4)	1.8 (16)	0.6 (5)	0.7 (6)	0.5 (4)	0.3 (3)	4.3 (38)
Subtotal	2010–2012 (*n* = 935)	64.4 (602)	17.9 (167)	12.8 (120)	4.0 (37)	1.0 (9)	0 (0)	0 (0)	0 (0)	0 (0)	4.9 (46)
	2013–2015 (*n* = 1053)	52.4 (552)	30.4 (320)	14.0 (147)	1.5 (16)	1.3 (14)	0.3 (3)	0.1 (1)	0 (0)	0 (0)	3.2 (34)
	2016–2018 (*n* = 1124)	49.2 (553)	37.3 (419)	12.2 (137)	0.4 (4)	0.6 (7)	0 (0)	0.1 (1)	0 (0)	0.1 (1)	1.2 (13)
	2019–2021 (*n* = 2020)	51.3 (1037)	26.4 (531)	18.3 (369)	0.9 (19)	1.1 (22)	0.5 (10)	0.7 (15)	0.4 (8)	0.5 (11)	4.1 (82)
	2022–2023 (*n* = 1429)	37.0 (529)	46.7 (667)	14.0 (200)	1.0 (14)	0.6 (9)	0.1 (1)	0.3 (4)	0.3 (4)	0 (0)	2.2 (31)
	Total (*n* = 6561)	49.9 (3273)	32.0 (2104)	14.8 (973)	1.4 (90)	0.9 (61)	0.2 (11)	0.3 (21)	0.2 (12)	0.2 (11)	3.1 (206)

The breakpoint of ciprofloxacin (≥1 µg/mL) was followed according to the Clinical and Laboratory Standards Institute (CLSI) (2023).

Although no resistant isolate was observed before 2013, a total of 1.7% and 0.6% of isolates showed MIC ≥8 µg/mL in 2019–2021 and 2022–2023, respectively (Table 1). The rate of resistance also differed among different animal species. Resistance was lower in pig isolates compared to the isolates obtained from other animals. Even though the resistance rate was 3.2% in cattle, all isolates showed MIC 1 µg/mL. Generally, the resistance rate was higher in chicken and duck isolates. Furthermore, the number of isolates showing an elevated level of MIC 8–≥16 µg/mL was higher in chickens (0.8%) and ducks (1.5%) compared to cattle (0%) and pigs (0.1%).

### 3.2. Distribution of ciprofloxacin-resistant Salmonella by serotypes

Ciprofloxacin-resistant *Salmonella* was observed in 20 serotypes (Table 2). The most frequently observed serotypes were *S.* Gallinarium (21.8%) and *S.* Typhimurium (21.8%), followed by *S.* Kentucky (11.2%), *S.* Albany (9.2%), *S.* Indiana (5.3%), and *S.* Montevideo (4.9%). Moreover, the level of MIC was different by serotype. Most serotypes showed MIC at 1–2 µg/mL, especially 100% of *S.* Gallinarium and 91.1% of *S.* Typhimurium isolates. Of note, six serotypes had high MIC (8 µg/mL), and very high MIC (≥16 µg/mL) was observed in *S.* Kentucky, *S.* Indiana, and *S.* Albany.

**Table 2.** Distribution of ciprofloxacin minimum inhibitory concentration (MIC) and resistance of *Salmonella* isolates obtained from food animals during 2010–2023 in South Korea.

Serotypes (No.)	Minimum inhibitory concentration of ciprofloxacin (µg/mL)					
	1	2	4	8	16	>16
*S.* Gallinarium (*n* = 45)	31 (34.4)	14	–	–	–	–
*S.* Typhimurium (*n* = 45)	29	12	4	–	–	–
*S.* Kentucky (*n* = 23)	–	–	–	11	11	1
*S.* Albany (*n* = 19)	4	5	4	5	1	–
*S.* Indiana (*n* = 11)	2	1	1	–	–	7
*S.* Montevideo (*n* = 10)	5	4	1	–	–	–
*S.* Hadar (*n* = 8)	–	7	–	1	–	–
*S.* Virchow (*n* = 6)	5	1	–	–	–	–
*S.* Enteritidis (*n* = 4)	4	–	–	–	–	–
*S.* Infantis (*n* = 6)	3	2	–	1	–	–
*S.* Agona (*n* = 5)	2	1	–	2	–	–
*S.* Rissen (*n* = 4)	–	4	–	–	–	–
*S.* Give (*n* = 4)	–	4	–	–	–	–
*S.* London (*n* = 2)	2	–	–	–	–	–
*S.* Senftenberg (*n* = 2)	–	2	–	–	–	–
*S.* Typhimurium variant (*n* = 2)	1	1	–	–	–	–
*S.* Emek (*n* = 1)	–	1	–	–	–	–
*S.* Muenchen (*n* = 1)	1	–	–	–	–	–
*S.* Reading (*n* = 1)	–	1	–	–	–	–
*S.* Seremban (*n* = 1)	–	–	–	1	–	–
Unidentified (*n* = 6)	1	1	1	–	–	3
Total (*n* = 206)	90 (43.7)	61 (30.0)	11 (5.3)	21 (10.2)	12 (5.8)	11 (5.3)

### 3.3. QRDR mutation and detection of qnr genes

All ciprofloxacin-resistant isolates showed mutations in *gyrA* and *parC* (Table 3). However, no mutation was found in *gyrB*. The most commonly observed mutations in *gyrA* were S83F (89.1%, 49/55), followed by D87N (40%, 22/55), D87G (20%, 11/55), D87Y (7.3%, 4/55) and S83Y (5.5%, 3/55). The predominant mutations identified in *parC* were T57S (89.1%, 49/55) and S80I (43.6%, 24/55). We found a stepwise pattern among the QRDR mutations. The number of mutation sites increases in high-MIC isolates. The MICs of ≥16 µg/mL isolates possessed mutations at 4–5 sites. In addition, mutation patterns were different by serotype. In *S.* Albany, the S83F mutation in *gyrA* and T57S in *parC* were common, while in *S.* Kentucky, S83F and D87N in *gyrA*, T57S and S80I in *parC,* and in *S.* Indiana, S83F and D87G in *gyrA*, T57S and S80R in *parC,* and L416F in *parE* were frequently detected.

**Table 3.** Distribution of plasmid-mediated quinolone resistance (PMQR) genes and quinolone resistance determining region (QRDR) mutations in *Salmonella* isolates obtained from food animals during 2010–2023 in South Korea.

CIP MIC (µg/mL) (No. of isolates)	Serotypes (No.)	*gyrA*	*gyrB*	*parC*	*parE*	PMQR gene (No.)
4 (*n* = 11)	*S.* Albany (*n* = 2)	S83F	WT	T57S, P112L	WT	*qnrB1* (*n* = 2)
	*S.* Albany (*n* = 2)	S83F	WT	T57S	WT	*qnrS* (*n* = 2)
	*S.* Indiana (*n* = 1)	D87N	WT	T57S	WT	*qnrB1* (*n* = 1)
	*S.* Montevideo (*n* = 1)	D87G	WT	T57S	WT	*qnrB1, aac(6′)-Ib-cr* (*n* = 1)
	*S.* Typhimurium (*n* = 3)	S83Y	WT	WT	WT	*qnrS* (*n* = 3)
	*S.* Typhimurium (*n* = 1)	D87Y	WT	WT	WT	*qnrS* (*n* = 1)
	Unidentified (*n* = 1)	S83F	WT	T57S	WT	*qnrS* (*n* = 1)
8 (*n* = 21)	*S.* Agona (*n* = 2)	S83F	WT	T57S	WT	*qnrS* (*n* = 2)
	*S.* Albany (*n* = 5)	S83F	WT	T57S	WT	*qnrS* (*n* = 5)
	*S.* Hadar (*n* = 1)	S83F	WT	T57S	WT	*qnrS* (*n* = 1)
	*S.* Infantis (*n* = 1)	S83F	WT	WT	WT	*qnrS* (*n* = 1)
	*S.* Kentucky (*n* = 9)	S83F, D87N	WT	T57S, S80I	WT	–
	*S.* Kentucky (*n* = 1)	S83F, D87Y	WT	T57S, S80I	WT	–
	*S.* Kentucky (*n* = 1)	S83F, D87Y	WT	S80I	WT	–
	*S.* Seremban (*n* = 1)	S83F, D87N	WT	T57S, S80I	WT	–
16 (*n* = 12)	*S.* Albany (*n* = 1)	S83F	WT	T57S	WT	*qnrS* (*n* = 1)
	*S.* Kentucky (*n* = 10)	S83F, D87N	WT	T57S, S80I	WT	–
	*S.* Kentucky (*n* = 1)	S83F, D87Y	WT	T57S, S80I	WT	–
>16 (*n* = 11)	*S.* Indiana (*n* = 6)	S83F, D87G	WT	T57S, S80R	L416F	*aac(6′)-Ib-cr* (*n* = 6)
	*S.* Indiana (*n* = 4)	S83F, D87G	WT	T57S, S80R	L416F	–
	*S.* Kentucky (*n* = 1)	S83F, D87N	WT	T57S, S80I	WT	*qnrS* (*n* = 1)

CIP, ciprofloxacin; MIC, minimum inhibitory concentration; WT, wild type; –, not detected.

Overall, all isolates with MIC ≥4 µg/mL carried the *qnr* gene. Moreover, a total of three PMQR genes (*qnrB1, qnrS,* and *aac(6′)-Ib-cr)* were detected (Table 3). Among them, *qnrS* (32.7%, 18/55) was predominantly found, followed by *aac(6′)-Ib-cr* (12.7%, 7/55) and *qnrB1* (7.3%, 4/55). In the serotype level, *qnrS* was mostly identified in *S.* Albany (8 isolates), *aac(6′)-Ib-cr* in *S.* Indiana (6 isolates), and *qnrB1* in *S.* Albany (2 isolates).

### 3.4. Molecular characterization

The high-level (MIC ≥4 µg/mL) ciprofloxacin-resistant isolates were distributed nationwide and obtained from 39 farms located in all provinces except one (Table 4). Overall, only one isolate was identified in each farm except one (A), which detected five isolates: four *S.* Kentucky and one *S.* Seremban. Since high-level ciprofloxacin-resistant *S.* Kentucky isolates were first detected in chickens in 2013, ciprofloxacin resistance was observed in various other serotypes, including *S.* Kentucky and *S.* Indiana, which were mainly observed in chickens, while *S.* Albany was mostly identified in ducks. The ciprofloxacin-resistant isolates also exhibited resistance to non-fluoroquinolone. Most of the isolates demonstrated resistance to three more antimicrobials, commonly ampicillin, sulfisoxazole, and tetracycline. Of note, three isolates were resistant to third-generation cephalosporin. These isolates carried *bla*_CTX-M-65_ in *S.* Kentucky from one duck and two chickens.

**Table 4.** Characteristics of ciprofloxacin-resistant *Salmonella* isolates obtained from food animals during 2010–2023 in South Korea.

Serotypes	Isolates	Year	Province	Farm ID	Source	MIC (µg/mL)		Non-fluoroquinolone resistance patterns	MLST	PFGE pattern
						Ciprofloxacin	Cefotaxime			
*S.* Kentucky (*n* = 23)	13-S03-57	2013	Incheon	AQ	Chicken feces	8	≤0.5	AMP, GEN, STR, FIS, TET	198	KX7KA8
	19-S03-03	2019	Gyeonggi	D	Chicken carcass	16	≤0.5	AMP, FIS, TET	198	KX1KA1
	19-S03-04	2019	Gyeonggi	E	Chicken carcass	8	≤0.5	AMP, FIS, TET	198	KX1KA1
	19-S03-05	2019	Gyeonggi	AK	Chicken carcass	16	≤0.5	AMP, FIS, TET	198	KX1KA1
	19-S03-06	2019	Gyeonggi	AK	Chicken carcass	8	≤0.5	AMP, FIS, TET	198	KX1KA1
	19-S03-12	2019	Gyeongbuk	K	Chicken carcass	16	≤0.5	AMP, FIS, TET	198	KX1KA1
	20-A02-18	2020	Chungbuk	T	Pig feces	8	≤0.5	AMP, FIS, TET	198	KX4KA4
	20-A03-19	2020	Chungbuk	AI	Chicken feces	8	≤0.5	AMP, FIS, TET	198	KX4KA4
	20-S03-14	2020	Gyeonggi	O	Chicken carcass	16	≤0.5	AMP, FIS, TET	198	KX1KA2
	20-S03-15	2020	Gyeonggi	Y	Chicken carcass	16	≤0.5	AMP, FIS, TET	198	KX1KA2
	20-S03-20	2020	Chungbuk	AJ	Chicken carcass	16	≤0.5	AMP, FIS, TET	198	KX4KA4
	20-S03-21	2020	Chungbuk	AG	Chicken carcass	8	≤0.5	AMP, FIS, TET	198	KX4KA1
	20-S03-22	2020	Chungbuk	S	Chicken carcass	8	≤0.5	–	198	KX6KA6
	21-A03-35	2021	Gyeonggi	A	Chicken feces	16	≤0.5	–	198	KX2KA1
	21-A03-36	2021	Gyeonggi	A	Chicken feces	8	≤0.5	–	198	KX2KA1
	21-A03-37	2021	Gyeonggi	A	Chicken feces	8	≤0.5	AMP, FIS, TET	198	KX1KA1
	21-A03-38	2021	Gyeonggi	A	Chicken feces	8	≤0.5	AMP, FIS, TET	198	KX1KA1
	21-A04-47	2021	Gyeongnam	V	Duck feces	>16	>8*	AMP, FEP, CTX, CAZ, CHL, FIS, TET, SXT	198	KX4KA5
	22-E03-52	2022	Jeonbuk	AD	Diseased chicken	8	>8*	AMP, FEP, CTX, CAZ, TET	198	KX5KA7
	22-E04-49	2022	Chungbuk	M	Diseased duck	16	≤0.5	AMP, FIS, TET	198	KX1KA3
	22-E04-50	2022	Chungbuk	M	Diseased duck	16	≤0.5	AMP, FIS, TET	198	KX1KA3
	22-E04-51	2022	Chungbuk	M	Diseased duck	16	≤0.5	AMP, FIS, TET	198	KX1KA3
	23-A03-54	2023	Chungnam	F	Chicken feces	16	>8*	AMP, FEP, CTX, CAZ, TET	198	–
*S.* Indiana (*n* = 11)	18-S04-01	2018	Chungbuk	AB	Duck carcass	>16	≤0.5	AMP, CHL, STR, FIS, TET	17	IX1IA4
	19-A03-08	2019	Gangwon	AC	Chicken feces	>16	≤0.5	AMP, CHL, GEN, STR, FIS, TET	17	IX1IA1
	19-A03-09	2019	Gangwon	AC	Chicken feces	>16	≤0.5	AMP, CHL, GEN, STR, FIS, TET	17	IX1IA1
	19-E03-10	2019	Chungbuk	H	Diseased chicken	>16	≤0.5	AMP, CHL, GEN, STR, FIS, TET	17	IX1IA3
	20-A03-29	2020	Chungnam	Z	Chicken feces	>16	≤0.5	AMP, CHL, GEN, STR, FIS, TET	17	IX2IA1
	20-A03-30	2020	Chungnam	U	Chicken feces	>16	≤0.5	AMP, CHL, GEN, STR, FIS, TET	17	IX3IA2
	20-A04-33	2020	Gyeongnam	X	Duck feces	4	≤0.5	FIS, SXT	17	IX5IA7
	20-S03-16	2020	Gyeonggi	AA	Chicken carcass	>16	≤0.5	AMP, CHL, GEN, STR, FIS, TET	17	IX1IA1
	20-S03-17	2020	Gyeonggi	AA	Chicken carcass	>16	≤0.5	AMP, CHL, GEN, STR, FIS, TET	17	IX1IA1
	20-S04-23	2020	Chungbuk	Q	Duck carcass	>16	≤0.5	–	17	IX6IA5
	21-A03-40	2021	Chungbuk	P	Chicken feces	>16	≤0.5	AMP, CHL, GEN, STR, FIS, TET	17	IX4IA6
*S.* Albany (*n* = 10)	19-A04-11	2019	Jeonnam	C	Duck feces	8	≤0.5	AMP, CHL, FIS, TET, SXT	292	AX4AA1
	20-A03-31	2020	Jeonnam	L	Chicken feces	4	≤0.5	CHL, FIS, SXT	292	AX5AA4
	20-A04-32	2020	Jeonnam	AE	Duck feces	8	≤0.5	AMP, CHL, FIS, TET, SXT	292	AX3AA3
	20-E04-24	2020	Chungbuk	I	Diseased duck	8	≤0.5	AMP, CHL, FIS, TET, SXT	292	AX1AA1
	20-E04-25	2020	Chungbuk	I	Diseased duck	8	≤0.5	AMP, CHL, FIS, TET, SXT	292	AX1AA1
	20-E04-26	2020	Chungbuk	AF	Diseased duck	4	≤0.5	AMP, CHL, FIS, TET, SXT	292	NDAA5
	20-E04-27	2020	Chungbuk	AF	Diseased duck	4	≤0.5	AMP, CHL, FIS, TET, SXT	292	AX1AA5
	20-E04-28	2020	Chungbuk	I	Diseased duck	16	≤0.5	AMP, CHL, FIS, TET, SXT	292	AX1AA1
	21-S04-42	2021	Jeonbuk	J	Duck carcass	4	≤0.5	AMP, CHL, FIS, SXT	292	AX2AA2
	21-E04-43	2021	Jeonnam	B	Diseased duck	8	≤0.5	AMP, CHL, FIS, TET, SXT	292	AX1AA1
*S.* Typhimurium (*n* = 4)	14-A03-58	2014	Gyeongbuk	AN	Chicken feces	4	≤0.5	AMP, CHL, GEN, STR, FIS, TET, SXT	50	–
	15-A03-60	2015	Jeonbuk	AP	Chicken feces	4	≤0.5	AMP, CHL, STR, FIS, TET, SXT	50	–
	15-S03-59	2015	Chungbuk	AO	Chicken carcass	4	≤0.5	AMP, CHL, GEN, STR, FIS, TET, SXT	50	–
	21-E02-34	2021	Gyeongbuk	AL	Diseased pig	4	1	AMP, CHL, STR, FIS, TET, SXT	8316	–
*S.* Agona (*n* = 2)	23-A03-55	2023	Jeonbuk	N	Chicken feces	8	≤0.5	AMP, CHL, TET	13	–
	23-S03-56	2023	Jeonbuk	N	Chicken carcass	8	≤0.5	AMP, CHL, TET	13	–
*S.* Hadar	22-S04-53	2022	Gyeongnam	W	Duck carcass	8	≤0.5	STR	33	–
*S.* Infantis	18-A03-02	2018	Gyeongbuk	AH	Chicken feces	8	≤0.5	AMP, CHL, STR, FIS, TET, SXT	ND	–
*S.* Montevideo	21-A03-44	2021	Gyeongbuk	R	Chicken feces	4	≤0.5	STR, FIS, TET, SXT	4	MX1MA1
*S.* Seremban	21-A03-39	2021	Gyeonggi	A	Chicken feces	8	≤0.5	AMP, FIS, TET	198	–
Unknown	21-A04-41	2021	Jeonbuk	G	Duck feces	4	≤0.5	AMP, CHL, FIS, SXT	292	–

AMP, ampicillin; CAZ, ceftazidime; CHL, chloramphenicol; CTX, cefotaxime; FEP, cefepime; FIS, sulfisoxazole; GEN, gentamicin; STR, streptomycin; TET, tetracycline; SXT, trimethoprim/sulfamethoxazole. *carried *bla*_CTX-M-65_. ND, not determined.

The MLST analysis revealed specific ST by serotypes. All *S.* Kentucky was assigned to ST198, *S.* Albany to ST292, and *S.* Indiana to ST17 (Table 4). At the animal species level, *S.* Kentucky ST198 and *S.* Indiana ST17 were prevalent among chickens, while *S.* Albany ST292 was predominantly found in ducks. However, PFGE with two restriction enzymes (*Xba*I and *Avr*II) digestion differentiated the same STs (Figure S1). A total of 26 unique pulsotypes were detected, comprising eleven types in *S.* Kentucky, eight in *S.* Indiana, and seven in *S.* Albany among the 55 ciprofloxacin-resistant *Salmonella* isolates. Overall, PFGE patterns were heterogeneous except for *S.* Kentucky. One PFGE type, KX1KA1, was observed in 30.4% of *S.* Kentucky isolates. Furthermore, identical ST and PFGE types were found in the same and different farms. The KX1KA1 pattern in *S.* Kentucky was mainly observed in farms AK (*n* = 2), A (*n* = 2), D (*n* = 1), E (*n* = 1), and K (*n* = 1); in *S.* Albany, AX1AA1 was frequently detected in farms I (*n* = 3) and B (*n* = 1); and in *S.* Indiana, IX1IA1 was detected in farms AC (*n* = 2) and AA (*n* = 2).

## 4. Discussion

This extensive, long-term investigation identified the high-level ciprofloxacin resistance in various serotypes, especially *S.* Kentucky, *S.* Indiana, and *S.* Albany, mainly from chicken sources. The major resistance mechanism is the mutation at QRDR. In addition, specific serotypes and clones *S.* Kentucky ST198, *S.* Indiana ST17, and *S.* Albany 292 were widely detected. Of note, three ciprofloxacin-resistant *S.* Kentucky carried the *bla*_CTX-M-65_.

Overall, ciprofloxacin resistance was detected in 3.1% of the isolates. The resistance rate of the isolates differed by the various collection year groups, animal species, and level of MICs. Isolates from food animals had varying levels of MIC for ciprofloxacin, consistent with findings from previous studies (Jiang et al. 2014; Bai et al. 2015). Noticeably, susceptibility was decreased compared to 10 years ago. Furthermore, high-level ciprofloxacin-resistant isolates (≥4 µg/mL) have been detected in food animals since 2013. The high incidence of ciprofloxacin resistance in *Salmonella* strains isolated from food animals was also found in China (14.8%) (Chen et al. 2021), Ukraine (6.3%) (Kozytska et al. 2023), and South Africa (8%) (Mthembu et al. 2019). In Korea, Sin et al. (2020) reported the occurrence of ciprofloxacin-resistant *Salmonella* (3.5%) in food-producing animals is consistent with our findings.

The level of ciprofloxacin resistance was different by serotypes in this study. The resistance level for MIC 1–2 µg/mL was mainly detected in serotypes *S.* Gallinarium (29.8%) and *S.* Typhimurium (27.2%). In note, high-level resistance (MIC 4–>16 µg/mL) was detected in *S.* Kentucky (41.8%), *S.* Albany (18.2%), and *S.* Indiana (14.5%). This finding concurs with previous studies showing that resistance levels can vary depending on the serotypes (Zhang et al. 2017; Abd El-Aziz et al. 2021; Awosile et al. 2023). The high prevalence of ciprofloxacin resistance in *S.* Gallinaiurm (26.9%) from chickens has been reported in South Korea, consistent with our findings (Seo et al. 2019). Furthermore, ciprofloxacin resistance was detected in 72.4% of *S.* Gallinaium in India (Bangera et al. 2019) and 48% of *S.* Typhimurium in Malaysia (Syed Abu Thahir et al. 2023) isolated from chickens, which is greater than our current findings. Moreover, it was shown that the majority of the *S.* Kentucky isolates recovered from poultry (71.4%) in 28 European Union (EU) member states demonstrated high-level ciprofloxacin resistance (EFSA and ECDC 2020). In addition, Zengfeng et al. (2022) found that 89.9% of the *S.* Indiana isolates from food-producing animals in China exhibited resistance to ciprofloxacin. Our previous studies also reported the occurrence of high-level ciprofloxacin resistance in *S.* Kentucky (Moon et al. 2021) and *S.* Indiana (Moon et al. 2021) isolated from chickens.

Moreover, this study reported ciprofloxacin resistance in other serotypes, including *S.* Albany, *S.* Infantis, *S.* Agona, and *S.* Seremban. The prevalence of ciprofloxacin-resistant *S.* Albany has recently emerged significantly in food animals (Kaichao et al. 2020). In addition, the emergence of high-level ciprofloxacin resistance in the serotypes *S.* Infantis and *S.* Agona has been increased in humans and food-producing animals, including chickens, pigs, and cattle (Kuang et al. 2018; Lai et al. 2023). Furthermore, although the ciprofloxacin-resistant *S.* Seremban is less frequent, it has also been reported in humans and food animals (Hendriksen 2010).

*Salmonella* isolates in this investigation were obtained from various sources, with commonly observed ciprofloxacin resistance in chickens. The high prevalence of ciprofloxacin-resistant *Salmonella* isolated from poultry is consistent with findings from previous investigations (Kim et al. 2019; Moon et al. 2021). The reason might be due to the increased consumption of fluoroquinolones in poultry, particularly with about 70% of the total used 35–40 tons of enrofloxacin annually in Korean livestock (Animal and Plant Quarantine Agency (APQA) 2022).

The resistance of fluoroquinolones in *Salmonella* was caused by mutations in the subunits constituting topoisomerase II (*gyrA* and *gyrB*) and IV (*parC* and *parE*) in the QRDRs (Abd El-Aziz et al. 2021). In this study, all resistant isolates had mutations in *gyrA,* and most of the resistant isolates possessed mutations in *parC.* Moreover, the mutations in *gyrA* were most commonly detected at S83F, D87N, and D87G, whereas the most frequent mutations in *parC* occurred at T57S and S80I. *Salmonella* showing resistance to ciprofloxacin typically possesses a minimum of two mutations in *gyrA*, in addition to mutations in other QRDRs (Abd El-Aziz et al. 2021). These additional mutations in the QRDR genes triggered the obtaining of a significant level of resistance to fluoroquinolones. Cao et al. (2017) demonstrated that the sites, types, and number of amino acid substitutions in QRDR genes varied among serovars, suggesting that resistance can differ regarding types and numbers of mutations in QRDRs. The L416F change was found in *parE* in our investigation, while L416F has previously been reported in *Salmonella* strains isolated from China (Yang et al. 2023).

In this study, all isolates with a MIC of ≥4 µg/mL possessed the *qnr* genes. In addition, different PMQR genes, *qnrS, qnrB1,* and *aac(6′)-Ib-cr*, have been detected. Moreover, the presence of the *qnr* genes differed by serotype. Most of the serotypes carried one or more *qnr* or *aac(6′)-Ib-cr* genes. Nonetheless, no *qnr* genes were detected in *S.* Kentucky. Consistent with this investigation, *qnrS* and *qnrB1* genes were detected in nontyphoidal *Salmonella* strains isolated from humans and food animals, including chickens, pigs, and cattle in China (Chen et al. 2024), Ethiopia (Eguale et al. 2017), and Romania (Colobatiu et al. 2015). The *qnr* genes were not associated with high-level resistance or with serotypes. However, their presence can complement other mechanisms to enhance the accumulation of the mutational target sites, leading to the selection of high-level resistance (Shaheen et al. 2021). It was shown that the occurrence of the *aac(6′)-Ib-cr* gene can complement the high-level quinolone resistance (Hooper and Jacoby 2015). Microorganisms that possess this gene may become more challenging to treat and pose a critical risk to human and animal health (Wong et al. 2014). A previous investigation reported that the acquisition of plasmids encoding *aac(6′)-Ib-cr* by *Salmonella* resulted in elevated ciprofloxacin MICs, and this resistance determinant was found to be responsible for a significant rise in ciprofloxacin resistance observed in human cases of salmonellosis (Lee et al. 2021). Moreover, this mobile genetic element, found in livestock globally, can be plasmid-borne or situated in a chromosome, indicating a high likelihood of spreading (Biao et al. 2022).

We found that ciprofloxacin-resistant *Salmonella* also showed resistance to other commonly used antimicrobials in livestock. Interestingly, most of them, except *S.* Kentucky, demonstrated resistance to ampicillin, sulfisoxazole, tetracycline, and chloramphenicol. This is consistent with the previous findings, which showed that ciprofloxacin-resistant *S.* Indiana strains obtained from chickens demonstrated multidrug resistance, including ampicillin, chloramphenicol, tetracycline, and trimethoprim-sulfamethoxazole (Bai et al. 2016). In addition, it was revealed that ciprofloxacin-resistant *S.* Indiana and *S.* Typhimurium isolated from food animals simultaneously exhibited resistance to these antimicrobials (Chen et al. 2024; Han et al. 2024). Moreover, concurring with the previous investigations, our research demonstrated that *Salmonella* strains recovered from humans and food-producing animals exhibited an identical resistance pattern with ampicillin, chloramphenicol, gentamicin, tetracycline, and trimethoprim/sulfamethoxazole (Wołkowicz et al. 2021; Borah et al. 2022).

It was observed that three ciprofloxacin-resistant isolates carrying *bla*_CTX-M-65_ in *S.* Kentucky from two chickens and one duck were detected in this study. The prevalence of *bla*_CTX-M-65_-carrying *Salmonella* in food animals, particularly chickens, has been documented worldwide, including in China (Lin et al. 2015), Australia (Sia et al. 2021), the EU (EFSA and ECDC 2020), and Korea (Kang et al. 2024). Moreover, *Salmonella* strains obtained from food animals and humans exhibit similar genetic characteristics of cephalosporin resistance (Chen et al. 2019). The development and transmission of the *bla*_CTX-M-65_ gene may exacerbate the cephalosporin resistance in human salmonellosis. Furthermore, the infection caused by ciprofloxacin and third-generation cephalosporin co-resistant *Salmonella* in individuals further aggravates the situation (Jiang et al. 2024).

The MLST analysis revealed the occurrence of several STs representing specific serotypes. Among them, *S.* Kentucky ST198 and *S.* Indiana ST17 were predominantly detected in chickens, while *S.* Albany ST292 was commonly identified in ducks. Previous studies reported the frequent prevalence of ciprofloxacin-resistant *S.* Kentucky ST198 clones in chickens in Korea (Moon et al. 2021), China (Xiong et al. 2020), and Spain (Samper-Cativiela et al. 2022). Furthermore, multidrug-resistant *S.* Albany ST292 has been detected in humans and food animals, posing human health hazards (Monte et al. 2019). Recently, ciprofloxacin-resistant *S.* Indiana ST17 has also frequently been identified in food-producing animals, including chickens and ducks (Wang et al. 2020; Du et al. 2022), which could be spread to humans and other animals, posing potential health problems.

The PFGE analysis detected a total of 26 unique pulsotypes, including novel types, among the ciprofloxacin-resistant *Salmonella* isolates. Several types were found in different farms located in various provinces throughout the country, possibly because of the extensive spread of these clones. Previous studies have shown that multidrug-resistant *Salmonella* clones are widely spread in both humans and food animals (Borah et al. 2022; Manzari et al. 2022). Our analysis revealed that several new clones emerged and have the potential to be transmitted to humans or other animals.

## 5. Conclusion

The present study provides important insights regarding the prevalence and mechanisms of ciprofloxacin-resistant *Salmonella* isolates obtained from food animals. Ciprofloxacin-resistant isolates commonly contained S83F and D87N mutations in *gyrA;* T57S and S80I in *parC*; and L416F in *parE* as well as carrying PMQR genes (*qnrB1, qnrS,* and *aac(6′)-Ib-cr)*. Moreover, particular serotypes and clones such as *S.* Kentucky ST198, *S.* Albany ST292, and *S.* Indiana ST17 were predominantly detected that could potentially contribute to the dissemination of fluoroquinolone-resistant *Salmonella* in livestock. In addition, this resistance can be transmitted to humans through direct contact or the food chain. Thus, the findings of this study suggest the importance of regular monitoring and strict regulation of antimicrobial use in food animals. Moreover, further research on vertical and horizontal antimicrobial resistance gene transmission is required to determine the potential health hazards for humans and other animals.

## Supplementary Material

Supplemental Material

## Data Availability

The data obtained in the present study are available within the article and supplementary materials.
