# Supplementary material for: Prevalence and characterization of ciprofloxacin-resistant Salmonella enterica spp. isolated from food animals during 2010–2023 in South Korea
Source: Vet Q. 2025 Mar 17;45(1):1–11. doi: 10.1080/01652176.2025.2473733 (PMC11915734; doi:10.1080/01652176.2025.2473733)
Supplement: Supplemental Material [file TVEQ_A_2473733_SM9883.docx]

**Supplementary**

Supplementary Figure 1. XbaI- and AvrII-digested pulsed-field gel electrophoresis band profiles (A) and combination pattern (B) of ciprofloxacin-resistant Salmonella strains (n = 44) isolated from food animals during 2010–2023 in South Korea.


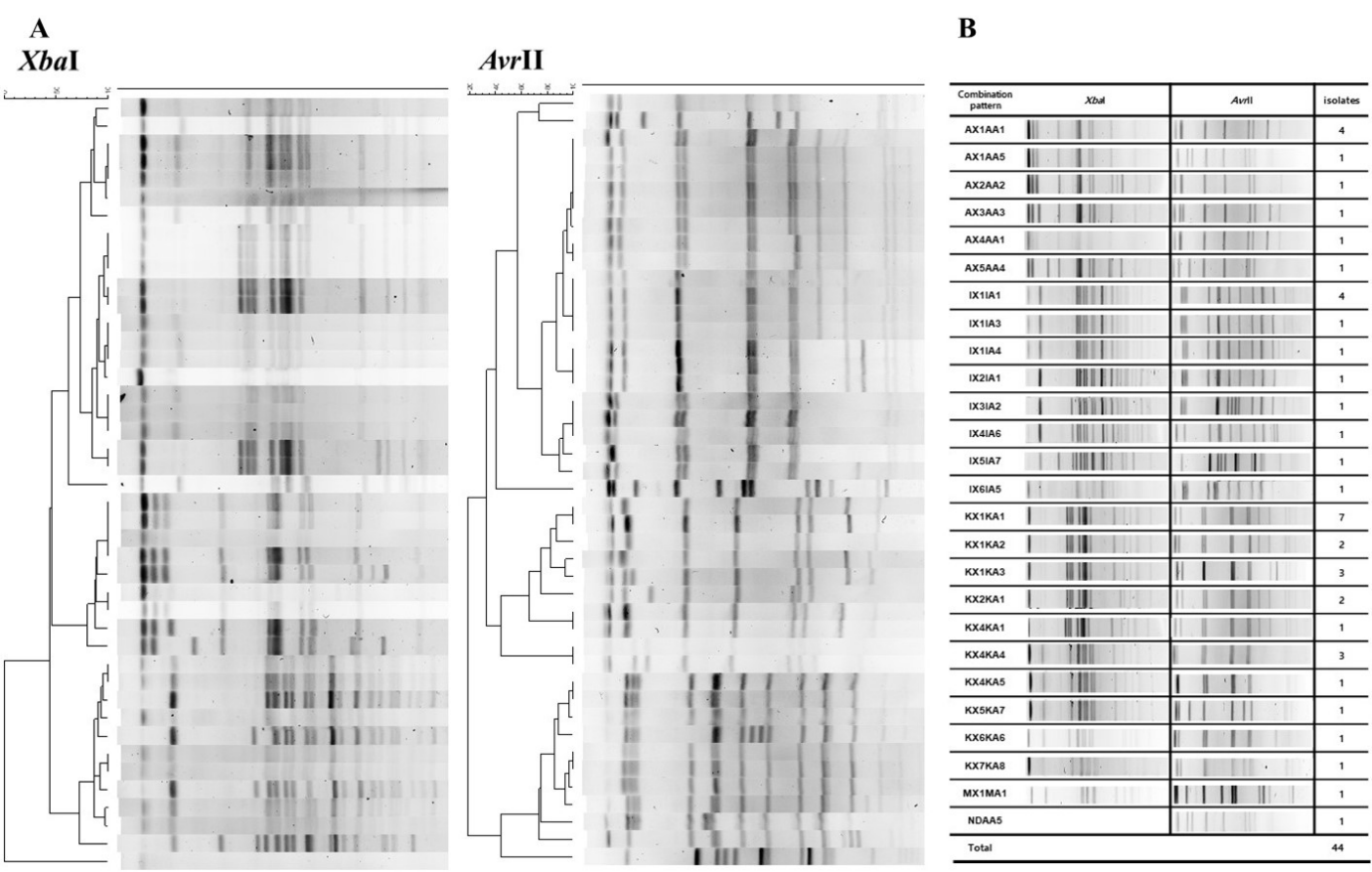


**Supplementary Table** 1. List of primer sequences and polymerase chain reaction (PCR) conditions

| Primer | Sequence (5'-3') | Size (bp) | PCR condition | References |
| --- | --- | --- | --- | --- |
| CTX-M-Universal^a^ | ATGTGCAGYACCAGTAARGTKATGGC | 593 | 35 cycles; 95℃ 1min + 60℃ 1min + 72℃ 1min | (Batchelor et al. 2005) |
|  | TGGGTRAARTARGTSACCAGAAYCAGCGG |  |  |  |
| CTX-M-9 families^a^ | GCTTTATGCGCAGACGAGTG | 703 | 35 cycles; 95℃ 1min + 60℃ 1min + 72℃ 1min | (Batchelor et al. 2005) |
|  | GCCAGATCACCGCAATATCA |  |  |  |
| CTX-M-9 families^a^ | GAAGCAGTCTAAATTCTTCGTGAAATAG | 1100 | 34 cycles; 94℃ 30sec + 60℃ 30sec + 72℃ 1min | (Saladin et al. 2002) |
|  | GGGCCAGTTGGTGATTTTGA |  |  |  |
| *gyrA* | CGA GAG AAA TTA CAC CGG TCA | 610 | 35 cycles; 94℃ 30sec + 60℃ 30sec + 72℃ 5min | (Kim et al. 2011) |
|  | AGC CCT TCA ATG CTG ATG TC |  |  |  |
| *gyrB* | CTG CTT TAC CAA CAA CAT TCC | 660 | 35 cycles; 94℃ 30sec + 60℃ 30sec + 72℃ 5min | (Kim et al. 2011) |
|  | TTG TCC GGG TTG TAC TCG TC |  |  |  |
| *parC* | ATG AGC GAT ATG GCA GAG C | 950 | 35 cycles; 94℃ 30sec + 60℃ 30sec + 72℃ 5min | (Kim et al. 2011) |
|  | GCG AAC AGA TGG TTC ATC AC |  |  |  |
| *parE* | GCG GAA GAT ATC TGG GAT CG | 897 | 35 cycles; 94℃ 30sec + 60℃ 30sec + 72℃ 5min | (Kim et al. 2011) |
|  | CAG CAG CAT ATC CAT CAT CG |  |  |  |
| *qnrB1* | GGC ATT GAA ATT CGC CAC T | 286 | 35 cycles; 94℃ 30sec + 60℃ 30sec + 72℃ 5min | (Kim et al. 2011) |
|  | TGG TCA GAT CGC AAT GTG TG |  |  |  |
| *qnrS* | GAC GTG CTA ACT TGC GTG AT | 380 | 35 cycles; 94℃ 30sec + 60℃ 30sec + 72℃ 5min | (Kim et al. 2011) |
|  | ACT TAA GTC TGA CTC TTT CAG |  |  |  |
| *aac(6')-Ib-cr* | TGA CCT TGC GAT GCT CTA TG | 508 | 35 cycles; 94℃ 30sec + 60℃ 30sec + 72℃ 5min | (Kim et al. 2011) |
|  | TTA GGC ATC ACT GCG TGT TC |  |  |  |

^a^ Primer sequences used in *bla*_CTX-M,_

**References**

Batchelor M, Threlfall EJ, Liebana E. 2005. Cephalosporin resistance among animal-associated *Enterobacteria*: a current perspective. Expert Rev Anti Infect Ther. 3(3):403–417. doi. 10.1586/14787210.3.3.403.

Kim K-Y, Park J-H, Kwak H-S, Woo G-J. 2011. Characterization of the quinolone resistance mechanism in foodborne *Salmonella* isolates with high nalidixic acid resistance. Int J Food Microbiol. 146(1):52–56. doi. 10.1016/j.ijfoodmicro.2011.01.037.

Saladin M, Cao VTB, Lambert T, Donay J-L, Herrmann J-L, Ould-Hocine Z, Verdet C, Delisle F, Philippon A, Arlet G. 2002. Diversity of CTX-M β-lactamases and their promoter regions from *Enterobacteriaceae* isolated in three Parisian hospitals. FEMS Microbiol Lett. 209(2):161–168. doi. 10.1111/j.1574-6968.2002.tb11126.x.
